# Spicy genes: mapping quantitative genomic regions and candidate genes for capsaicinoid and capsinoid biosynthesis in pepper

**DOI:** 10.3389/fpls.2026.1823752

**Published:** 2026-06-15

**Authors:** Edoardo Vergnano, Matteo Martina, Peter Poláček, Yury Tikunov, Luciana Gaccione, Lorenzo Barchi, Arnaud Bovy, Ezio Portis

**Affiliations:** 1 Department of Agricultural, Forest and Food Sciences (DISAFA), Plant Genetics, University of Turin, Grugliasco, Italy; 2 Plant Breeding, Wageningen University & Research WUR, Wageningen, Netherlands

**Keywords:** candidate genes, capsaicinoid, *Capsicum*, capsinoids, QTLs

## Abstract

Capsaicinoids, the molecules responsible for pungency in pepper (*Capsicum* spp.), and their non-pungent analogs, capsinoids, are synthesized through the interaction of two distinct metabolic pathways: the branched chain fatty acid pathway and the phenylpropanoid pathway. These two families of bioactive compounds are unique to the genus Capsicum and, besides their importance for pepper taste, are associated with several beneficial effects, such as weight management, antioxidant activity and prevention of various diseases. Although QTLs associated with capsaicinoid and capsinoid accumulation have been reported in several studies, these findings remain dispersed across different populations, limiting their direct comparison and practical use in breeding. In this study, we aim to collect, compare, integrate, and synthesize the available literature on capsaicinoid and capsinoid QTLs. A total of 155 QTLs associated with these traits were physically mapped onto the reference pepper genome (CM334 -v1.6) and analyzed within a common genomic framework. The physical integration of the selected regions allowed us to identify 23 Quantitative Genomic Regions (QGRs) and prioritize potential candidate genes located within them. This genome-based integration advances beyond previous descriptive summaries, providing a unified physical framework for comparing QTLs across studies and genetic backgrounds. This review provides a comprehensive resource for researchers aiming to understand the genetic mechanisms behind capsaicinoid and capsinoid biosynthesis and for breeders focused on improving the levels of these bioactive compounds in pepper. It enables the identification of key QTL regions through the integration of data from diverse populations, highlights potential donor genotypes reported in the literature for specific traits, and facilitates the discovery of candidate genes for future functional validation and marker-assisted breeding. Overall, this study provides a consolidated genomic framework for understanding the genetic architecture of capsaicinoid and capsinoid biosynthesis and for accelerating the development of pepper cultivars with improved profiles of these bioactive compounds.

## 1 Introduction

Pepper (*Capsicum* spp.) is among the most important cultivated species worldwide. With a production of 42 million tons (37 million as fresh, 5 million as dry), pepper is the fourth most produced solanaceous crop after potatoes, tomatoes, and eggplants. China is the leading worldwide producer, followed by Mexico, Turkey, Indonesia, and Spain (FAOSTAT, 2024).

It belongs to the Solanaceae family, which includes roughly 3,000-4,000 species, classified into about 90 genera (Tripodi et al., 2021; Liu et al., 2025). This family is extremely heterogeneous and includes ornamental species, such as *Petunia hybrida, Schizanthus pinnatus*, medicinal plants (i.e *Nicotiana tabacum*, *N. rustica*, *Atropa belladonna*, *Mandragora officinarum*, *Datura stramonium)*, annual herbaceous species, perennial trees, as well as horticultural crops *(Solanum lycopersicum*, *S. tuberosum*, *Capsicum* spp., and *S. melongena*) (Knapp et al., 2004; Gebhardt, 2016). *Capsicum* spp. is highly heterogeneous, as the *Solanaceae* family itself, in terms of plant morphology, fruit shape, colors and organoleptic characteristics.

This genus is composed by 43 different species, five of which (*C. annuum*, *C. baccatum*, *C. chinense, C. frutescens*, and *C. pubescens*) were domesticated, cultivated, and consumed in various regions of the world (Carrizo García et al., 2016; Tripodi and Kumar, 2019; Barboza et al., 2022; Liu et al., 2023; Martina et al., 2025). The significant biodiversity that characterizes these five economically relevant species seems to be related to the species centers of domestication: *C. baccatum, C. pubescens*, and *C. chinense* appeared in South America, while *C. annuum* and *C. frutescens* in Mesoamerica (Pickersgill, 2007).

The *Capsicum* genus includes hot and sweet peppers, which are used in the food industry and in traditional medicine, due to the presence of numerous bioactive molecules (Hernández-Pérez et al., 2020). One of the primary characteristics of the hot pepper lies in its spiciness, making it a key spice in numerous traditional recipes. The molecules responsible for this pungent sensation are capsaicin and its analogues, collectively called capsaicinoids, which are produced only in *Capsicum* spp. These compounds are primarily synthesized in the pericarp and placenta of *Capsicum* fruits (Vázquez-Espinosa et al., 2023) and, under natural conditions, the function of capsaicinoids is to selectively target vertebrates for seed dispersal. In particular, these molecules repel mammals but not birds, thereby promoting long-distance seed dispersal (Tewksbury and Nabhan, 2001).

More than 22 capsaicinoids are known (Luján-Méndez et al., 2023), however, only five molecules are consistently present in pepper fruits: capsaicin, dihydrocapsaicin, nordihydrocapsaicin, homocapsaicin, and homodihydrocapsaicin (**Table 1**). Among these, capsaicin and dihydrocapsaicin together constitute approximately 80-90% of the total capsaicinoid content in the fruits (Contreras-Padilla and Yahia, 1998).

**Table 1:** Capsaicinoid and capsinoid structure, molecular formula (MF), molecular weight (MW), and in model organism proved properties, adapted from Luo et al., 2011.

Name	MF	Chemical structure	MW (g/mol)	Properties	References
Capsaicin (trans-8-methyl-N-vanillyl-6-nonenamide)	C_18_H_27_NO_3_	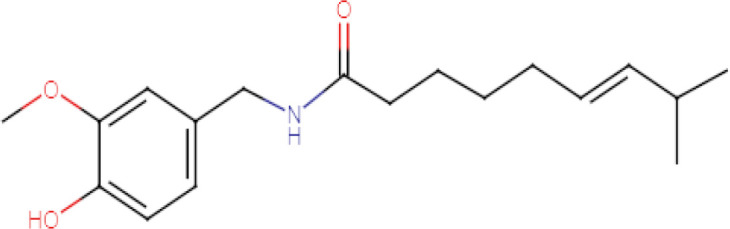	305.4	Analgesia, anticancer, anti-inflammation, antioxidant, anti-obesity	(Knotkova et al., 2008; Yang et al., 2010; Janssens et al., 2014)
Dihydrocapsaicin (8-methyl-N-vanillyl-nonanamide)	C_18_H_29_NO_3_	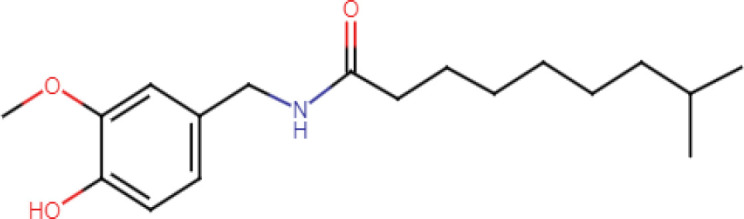	307.4	Analgesia, anticancer, anti-inflammation, antioxidant, anti-obesity	(Choi et al., 2010; Xie et al., 2016; Lee et al., 2019)
Nordihydrocapsaicin (7-methyl-N-vanillyl-octamide)	C_17_H_27_NO_3_	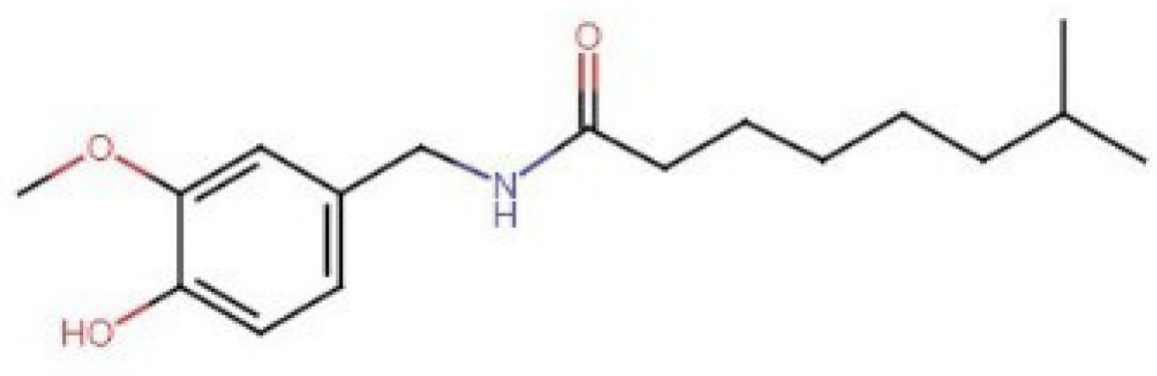	293.4	anti-obesity	(Janssens et al., 2014)
Homodihydrocapsaicin (9-methyl-N-vanillyl-decamide)	C_19_H_31_NO_3_	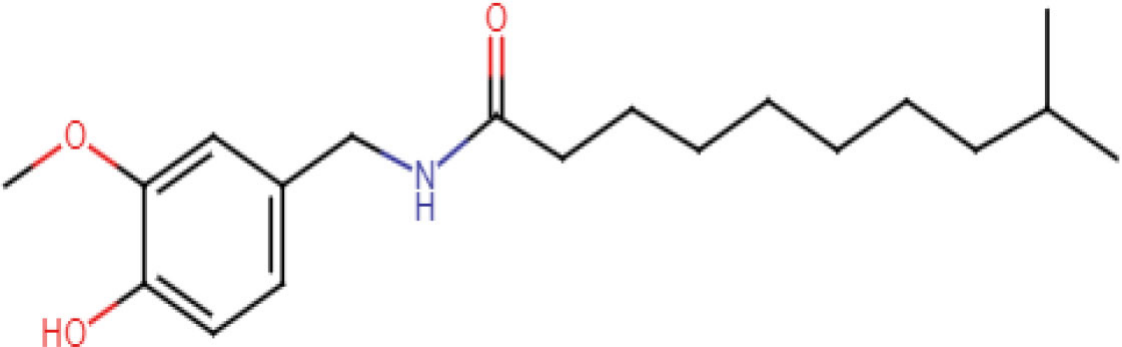	321.5		
Homocapsaicin (trans-9-methyl-N-vanillyl-7-decenamide)	C_19_H_29_NO_3_	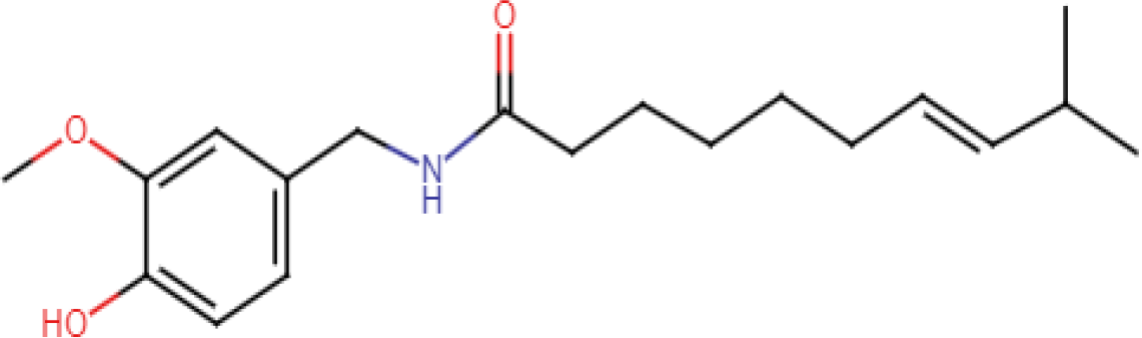	319.4		
Capsiate (8-methyl-6-E-nonenoic acid)	C_18_H_26_O_4_	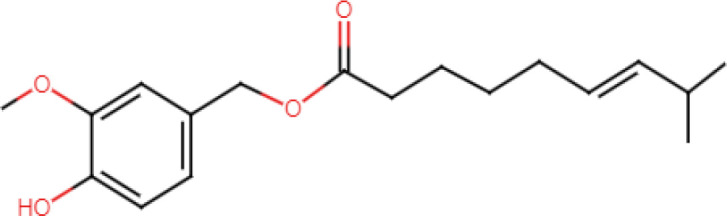	306.4	Anticancer, anti-inflammation, antioxidant, anti-obesity	(Pyun et al., 2008; Lee et al., 2010)
Nordihydrocapsiate (4-Hydroxy-3-methoxybenzyl 7-methyloctanoate)	C_17_H_26_O_4_	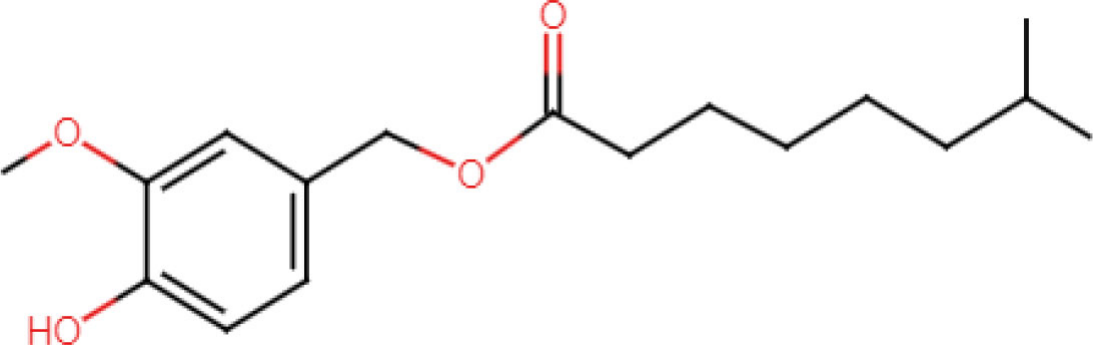	294.4	Anticancer anti-inflammation	(Friedman et al., 2018, 2019)
Dihydrocapsiate (4-Hydroxy-3-methoxybenzyl 8-methylnonanoate)	C_18_H_28_O_4_	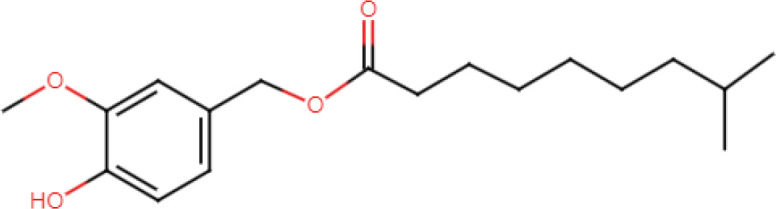	308.4	Anticancer, anti-inflammation, antioxidant, anti-obesity	(Baboota et al., 2018)

Another peculiar biochemical class reported in peppers (both sweet and spicy varieties) is capsinoids. This class, discovered in 1989 in *C. annuum* (CH-19 Sweet) fruits (Kobata et al., 1998), comprises capsiate, dihydrocapsiate, and nordihydrocapsiate, and is chemically very similar to the capsaicinoids class. In fact, capsinoids and capsaicinoids possess analogous structural properties, with the primary difference lying in their central ester bond (**Table 1**). Capsinoids consist of vanillyl alcohol esterified with fatty acids, whereas capsaicinoids are composed of vanillylamine linked to various branched-chain fatty acids through amide bonds (Sasahara et al., 2010; Luo et al., 2011; Tanaka, 2025). Compared to capsaicinoids, capsinoids offer a milder sensory effect when consumed and lack pungency entirely (Silvester et al., 2019). However, this difference does not diminish the importance of this class from a pharmacological standpoint. Both chemical classes are characterized by various beneficial impacts on human health, such as analgesic action, antioxidant, *in vitro* anti-cancer activity and suppression of body fat accumulation, making them extremely intriguing molecules from a medical perspective (Macho et al., 2003; Tremblay et al., 2016; Zhang et al., 2020; Thongin et al., 2022). Capsaicinoid and capsinoid biosynthesis depends on the interaction between the phenylpropanoid and branched-chain fatty acid pathways and is regulated by both structural and regulatory genes. Previous studies have identified key genes involved in pungency and capsaicinoid accumulation, including *Pun1*, *pAMT*, *CaMYB108*, and *MYB31* (Stewart et al., 2005; del Rosario Abraham-Juárez et al., 2008; Arce-Rodríguez and Ochoa-Alejo, 2017; Zhu et al., 2019). Genetic mapping and QTL analyses have also identified several genomic regions associated with capsaicinoid and capsinoid variation. However, these QTLs were reported across different populations, marker systems, phenotyping methods, and pepper genome references, making direct comparison difficult and limiting their use in breeding. To address this gap, this review collects and integrates published QTLs for capsaicinoid and capsinoid-related traits into a common genomic framework. This approach allowed the identification of Quantitative Genomic Regions (QGRs), the prioritization of candidate genes, and the development of a unified resource to support future functional studies and marker-assisted breeding for improved capsaicinoid and capsinoid profiles in pepper.

## 2 Capsaicinoid and capsinoid metabolic pathway

Understanding the metabolic and regulatory pathways underlying capsaicinoid and capsinoid biosynthesis is essential for interpreting QTL and QGR results. Genes belonging to these pathways provide biologically meaningful candidates when they co-localize with QTLs or QGRs associated with capsaicinoid and capsinoid variation. Thus, the integration of biosynthetic pathway information, structural gene variation, and transcriptional regulation provides a functional basis for candidate gene discovery within the QGR framework used in this review. Structural genes may explain major changes in pungency or capsinoid production, whereas regulatory genes may contribute to quantitative variation in metabolite accumulation through their effects on pathway gene expression. When these genes are located within QGRs, they represent strong candidates for marker development and marker assisted selection. In breeding programs, allelic variation in major structural genes can be exploited to develop pungent, low-pungency, or non-pungent cultivars, whereas regulatory genes may provide targets for fine-tuning capsaicinoid and capsinoid levels.

### 2.1 Biosynthetic pathways of capsaicinoids and capsinoids

**Figure 1:**
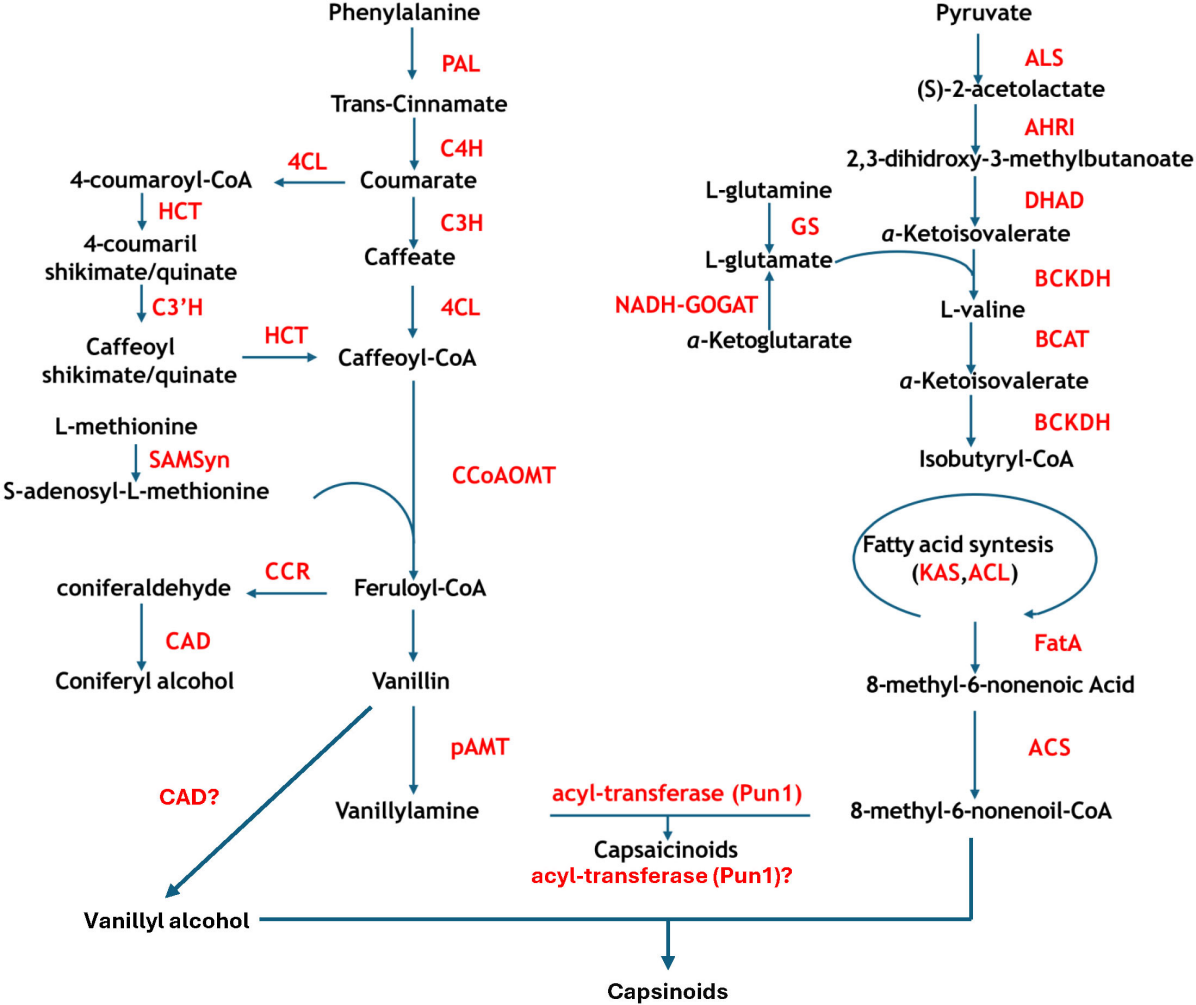
Overview of capsaicinoids and capsinoids metabolic pathway adapted from (Arce-Rodríguez and Ochoa-Alejo, 2019; Barros et al., 2019; Venkatesh et al., 2023).

Capsaicinoids are synthesized through the interaction of two distinct metabolic pathways: the phenylpropanoid pathway and the branched chain fatty acid pathway (Bennett and Kirby, 1968; Kaiser et al., 2017). In the phenylpropanoid pathway (**Figure 1**), numerous enzymes are involved to synthesize vanillylamine.

The first key enzyme in this pathway is phenylalanine ammonia-lyase (PAL), which catalyzes the synthesis of cinnamic acid from phenylalanine. This initial reaction is followed by several catalytic processes involving seven different enzymes: cinnamate 4-hydroxylase (C4H), 4-coumarate CoA ligase (4CL), hydroxycinnamoyl transferase (HCT), coumaroyl shikimate 3′-(C3’H), coumarate 3-hydroxylase 3-hydroxylase (C3H), caffeoyl-CoA 3-O-methyltransferase (CCoAOMT), and finally, a putative aminotransferase (pAMT), which catalyzes the formation of vanillylamine from vanillin (Aza-González et al., 2011; Kabita et al., 2019).

In the branched-fatty-acid synthesis pathway (**Figure 1**), several enzymes are involved in the conversion of pyruvate to 8-methyl-6-noneoyl-CoA. These enzymes include acetolactate α-synthase (ALS), acetohydroxyacid reductoisomerase (AHRI), dihydroxy acid dehydratase (DHAD), branched-chain amino acid transferase (BCAT), branched-chain α-ketoacid dehydrogenase/decarboxylase (BCKDH), ketoacyl-ACP synthase (KAS), acyl carrier protein (ACP), ATP citrate lyase (ACL), acyl-ACP thioesterase (fatA), and acyl-CoA synthetase (*ACS*) (del Rosario Abraham-Juárez et al., 2008; Arce-Rodríguez and Ochoa-Alejo, 2019). Finally, vanillylamine and 8-methyl-6-noneoyl-CoA, are combined through the catalytic action of an acyl-transferase (*Pun1*) to form capsaicinoids (Stewart et al., 2005).

### 2.2 Key structural genes and allelic variation

Most of the genes encoding the biosynthetic enzymes of these two pathway branches were cloned and functionally confirmed in other species, such as tomato and Arabidopsis. For each of these, direct pepper orthologs are known, though their function has not been functionally validated in most cases, due to the recalcitrance of pepper to genetic transformation and regeneration (**Supplementary Table 1**).

The capsinoid biosynthetic pathway largely overlaps with the capsaicinoid biosynthetic pathway, sharing most of the same enzymes. However, it is distinguished by the presence of different mutations in the putative amino transferase (*pAMT*) gene, resulting in the suppression of vanillylamine production. Recent evidences support the hypothesis that the cinnamyl alcohol dehydrogenase (CAD) is responsible for the reduction of vanillin to vanillyl alcohol (precursor of capsinoids) in cases where the *pAMT* gene is not functional (Sano et al., 2022; Venkatesh et al., 2023).

The first mutated *pAMT^1^* gene was identified in the *C. annuum* accession CH-19 Sweet, a mutant derived from CH-19. This accession showed a T insertion at position 1291bp of the open reading frame (ORF), resulting in the formation of a premature TGA stop codon (Lang et al., 2009).

Subsequently, numerous mutations in the *pAMT* gene were identified in various accessions. For instance, in the *C. annuum accession* Himo (*pAMT^2^*), a mutation characterized by a T → C substitution at 775bp of the protein-coding region was discovered. This nucleotide change resulted in the substitution of cysteine at position 259 with arginine (Tanaka et al., 2010a).

Similarly, three alleles (*pAMT^3^*, *pAMT^4^*, and *pAMT^5^*), were discovered in *C. chinense* cultivars. The *pAMT^3^* variant, found in accession ‘Belize Sweet’, is characterized by a 5-bp insertion (TGGGC) in the eighth exon, which results in a frameshift mutation. The *pAMT^4^* form in cultivar ‘Zavory Hot’ is marked by an insertion of 2.3 kb due to a transposable element of the hAT super family in the fifth intron. Finally, the *pAMT^5^* variant identified in ‘Aji Dulce Strain 2’, exhibits an 8-bp insertion in the sixth exon, caused by a transposable element from the hAT superfamily (Tanaka et al., 2010b).

Two additional forms of the pAMT enzyme associated with capsinoids production have been identified, both resulting from the insertion of transposable elements belonging to the hAT superfamily. The first variant, named *pAMT^6^*, contains a 7-bp insertion (CTTTACT) in the second exon of the ORF and was discovered in the *C. chinense* accession No. 80 (Koeda et al., 2014). The second variant, designated *pAMT^7^*, exhibits a 2.8 kb insertion, also located in the second exon, and was identified in the *C. chinense* variety LP6 (Tanaka et al., 2015). Recently, six additional variants of the *pAMT* gene have been identified in pepper. These variants are named *pAMT8*, *pAMT9*, *pAMT10*, *pAMT11*, *pAMTL1*, and *pAMTL2*. *pAMT8* and *pAMT9* are both characterized by the presence of INDELs that result in loss of function. Specifically, *pAMT8* features a 12 bp deletion (TCTGCTGGTCTC) in the seventh exon, while *pAMT9* carries a 7 bp insertion (TCGGTAC) in the sixteenth exon. *pAMT10* is characterized by a SNP in the eleventh exon that introduces a premature stop codon, leading to a loss of gene function. Similarly, *pAMT11* is characterized by a 7 bp insertion (AATCAAG) in the eighth exon, also causing a loss of function (Park et al., 2015; Tanaka et al., 2015; Tsurumaki and Sasanuma, 2019; Yi et al., 2022). The last two alleles (*pAMTL1* and *pAMTL2*) were identified in two *C. chinense* cultivars. The *pAMTL1* allele contains a 2.3 kb transposon insertion located 591 bp upstream of the fourth exon, while *pAMTL2* features a similar 2.3 kb transposon insertion located 342 bp upstream of the fourth exon (Tanaka et al., 2019). Unlike the previous alleles, these do not result in complete loss of function but in a reduced production of the amino transferase enzyme. Overall, *pAMT^1^* to *pAMT^11^*, *pAMT^L1^* and *pAMT^L2^* are alleles of the same gene; *pAMT^1^* to *pAMT^11^* have been identified as loss-of-function variants, whereas *pAMT^L1^* and *pAMT^L2^* are “leaky” alleles that retain partial function and therefore reduce, rather than abolish*, pAMT* activity and aminotransferase production (Tanaka, 2025). Importantly, mutant *pAMT* alleles are recurrently found in capsinoid-related, low-pungency germplasm, and many of the corresponding accessions have been directly reported to accumulate capsinoids. In addition, *pAMT* has been functionally validated through virus-induced gene silencing (VIGS), which confirmed its role in vanillylamine formation and capsaicinoid biosynthesis (del Rosario Abraham-Juárez et al., 2008).

The other key gene in the capsaicinoid pathway*, Pun1* (also known as *AT3*), which encodes a BAHD acyltransferase; is represented by multiple independent *Pun1* loss-of-function alleles described across domesticated *Capsicum* (Tanaka, 2025). *Pun1* has also been functionally validated through VIGS, which confirmed its role in the final acyltransferase step of capsaicinoid biosynthesis (Stewart et al., 2005). The first *Pun1* loss-of-function allele was characterized in non-pungent *C. annuum* germplasm, where a 2.5 kb deletion removes 1.8 kb of the putative promoter and 0.7 kb of exon 1, abolishing the function of the acyltransferase required for capsaicinoid biosynthesis (*pun1¹*) (Stewart et al., 2005). Subsequently, additional independent *Pun1* mutant alleles were discovered, including *pun1²* in *C. chinense* (4-bp deletion in exon 1 causing a frameshift and early stop codon) (Stewart et al., 2007), *pun1³* in *C. frutescens* (premature stop codon leading to truncation in exon 2) (Stellari et al., 2010), *pun1^4^* in the *cultivar* ‘Nara Murasaki’ (single nucleotide insertion in exon 2 causing a frameshift) (Kirii et al., 2017), and *pun1^5^* in ‘Sampo Oamanaga’ (a large deletion spanning a genomic region including *Pun1*) (Moe et al., 2024). Together, *pun1¹–pun1^5^* constitute a set of loss-of-function alleles that underpins the on/off inheritance of pungency across diverse *Capsicum* genetic backgrounds (Egan et al., 2019).

### 2.3 Transcriptional regulation and regulatory networks

In addition to biosynthetic enzymes, transcription factors play an important role in regulating gene expression within the capsaicinoid biosynthetic pathway. These transcription factors belong to various gene families, including MYB, bHLH, WRKY, and ERF (Arce-Rodríguez and Ochoa-Alejo, 2017; Song et al., 2020; Liu et al., 2021; Zhang et al., 2023a). Most of them are responsive to a wide range of phytohormones, such as indoleacetic, jasmonic, salicylic, and gibberellic acids, as well as ethylene. Furthermore, environmental stimuli including wounding, temperature fluctuations, and light exposure also influence the expression of these transcription factors, suggesting that capsaicinoid biosynthesis is modulated by a complex network integrating both developmental cues and external environmental signals (Arce-Rodríguez and Ochoa-Alejo, 2017).

The regulatory network controlling capsaicinoid biosynthesis appears to involve coordinated interactions among different transcription factor families. MYB transcription factors act as central regulators by controlling the expression of structural genes in the capsaicinoid pathway (Arce-Rodríguez and Ochoa-Alejo, 2017; Zhu et al., 2019; Sun et al., 2020), while bHLH proteins may cooperate with MYB factors through protein–protein interactions to modulate transcriptional activation (Villa-Rivera and Ochoa-Alejo, 2021). WRKY transcription factors can further enhance this regulatory module by influencing MYB-dependent expression patterns (Zhu et al., 2019; Zhang et al., 2023a), whereas ERF transcription factors may connect ethylene- and stress-related signaling with the regulation of capsaicinoid biosynthetic genes (Song et al., 2020, 2025; Wen et al., 2022). Together, these transcription factor families suggest that capsaicinoid accumulation is controlled by an integrated regulatory network rather than by isolated regulators. *MYB31* is considered the primary transcription factor regulating capsaicinoid biosynthesis. Initially identified in the cultivar ‘Tampiqueño 74’, this transcription factor contains an R2R3-type DNA-binding domain that enables it to bind the promoters of several key genes involved in the capsaicinoid biosynthetic pathway, including *Comt*, *BCAT*, *Kas1*, *pAMT*, and *AT3*, thereby functioning as a master regulator of their expression (Arce-Rodríguez and Ochoa-Alejo, 2017). Multiple studies have shown that *MYB31* expression is localized specifically in the fruit placenta and its expression levels are strongly correlated with capsaicinoid content (Zhu et al., 2019; Chen et al., 2024).

In addition to *MYB31*, at least ten other transcription factors belonging to the R2R3-MYB superfamily, *CaMYB108*, *CaMYB48*, *CaMYB47*, *CaMYB64*, *CaMYB73*, *CaMYB74*, *CaMYB87*, *CaMYB92*, *CaMYB103*, and *CaMYB115* have been identified through co-expression analyses as candidate regulators of capsaicinoid accumulation in the fruit (Borevitz et al., 2000; Sun et al., 2019, 2020; Wang et al., 2020). Notably, virus induced gene silencing (VIGS) has been performed on *CaMYB31*, *CaMYB48* and *CaMYB108*, leading to reduced expression of capsaicinoid biosynthetic genes. These findings highlight the central role of MYB-type transcription factors in modulating capsaicinoid biosynthesis in pepper (Arce-Rodríguez and Ochoa-Alejo, 2017; Zhu et al., 2019; Sun et al., 2020). Another transcription factor related to the MYB family is *CaDIV14*, a *DIVARICATA-like gene* whose expression is positively correlated with *MYB31* and *AT3*, suggesting a potential role in promoting capsaicinoid accumulation in pepper (Arce-Rodríguez et al., 2021).

The basic helix-loop-helix (bHLH) family includes regulators that often interact with MYB transcription factors (Zimmermann et al., 2004; Pireyre and Burow, 2015). In pepper, genome-wide studies have identified *CabHLH007*, *CabHLH009*, *CabHLH026*, *CabHLH063*, and *CabHLH086* as candidate regulators of capsaicinoid biosynthesis (Liu et al., 2021). Their expression levels correlate with capsaicinoid content in the placenta, and yeast two-hybrid assays confirmed their interaction with *MYB31*, supporting a cooperative role in activating biosynthetic genes (Villa-Rivera and Ochoa-Alejo, 2021).

Another transcription factor that cooperates with *MYB31* in promoting the expression of genes involved in capsaicinoid biosynthesis is *WRKY9*, a member of the WRKY transcription factor family. Identified in *C. chinense*, VIGS-based validation suggested the role of WRKY9 as *MYB31* expression enhancer in placenta tissue, potentially explaining the average higher pungency observed in some *C. chinense* cultivars (Zhu et al., 2019). In addition, genome-wide analyses have identified other WRKY transcription factors, such as *WRKY25*, which may influence capsaicinoid production indirectly by regulating upstream genes in the phenylpropanoid pathway (Zhang et al., 2023a).

*AP2/ERF* transcription factors, acting downstream of ethylene and having a wide range of functions in plant development and stress responses, are also found to regulate a number of genes in capsaicinoids production, one of the ethylene-responsive transcriptional factors, *CcERF2* has been reported to function as a key regulator of capsaicinoid biosynthesis (Wen et al., 2022). Moreover, other ERF-type transcription factors such as *CaERF92, CaERF102, CaERF111*, and *CaERF53* show expression patterns consistent with the degree of capsaicinoid accumulation, particularly when plants are challenged with heat, suggesting a possible connection between the environmental cues and the pungency metabolism in pepper (Song et al., 2020). Notably, VIGS-mediated silencing of *CaERF102* or *CaERF111* led to downregulation of capsaicinoid biosynthetic genes and a strong decrease in capsaicin and dihydrocapsaicin levels (Song et al., 2025). In summary, for only a handful transcription factors, their role in regulating capsaicinoid content has been functionally validated in pepper through VIGS (*CaMYB31, CaMYB48, CaMYB108, CcWRKY9, CaERF102 and CaERF111*), while many others are considered candidate regulators of the capsaicinoid pathway, based on their spatio-temporal expression, co-expression analysis, or transcription factor interaction studies, such as yeast 2 hybrid analysis.

## 3 Health effects of capsaicinoids and capsinoids

Capsaicinoids and capsinoids offer a variety of health benefits and have attracted growing interest from scientists in recent years (Adetunji et al., 2022). Their biological relevance, together with their role in pepper quality and consumer perception, makes them important target traits for genetic studies and breeding programs. For this reason, understanding the genomic regions and candidate genes controlling their accumulation is central to the objective of this review. However, the strength of the evidence supporting their health-related effects differs depending on the biological effect considered and on the experimental model used. Therefore, findings from *in vitro* assays, animal studies, and human interventions should be interpreted separately.

### 3.1 Metabolic effects

Capsaicinoids and capsinoids have thermogenic properties, which increase energy consumption and fat oxidation. Therefore, they can potentially aid weight management and reduce the risk of obesity in humans (Whiting et al., 2012; Snitker et al., 2009; Rogers et al., 2018; Zhang et al., 2023b). These compounds interact with TRPV1 (transient receptor potential vanilloid 1), which modulates body temperature and energy homeostasis. When TRPV1 is activated, both capsaicinoids and capsinoids stimulate thermogenesis and lipid oxidation, which may contribute to body fat reduction and weight loss (Whiting et al., 2012). With regular intake, they may also increase daily energy expenditure by around 50 kcal, which can contribute to clinically significant weight loss over time. Moreover, both compounds promote the formation of brown adipose tissue (Kida et al., 2018).

### 3.2 Cardiovascular, gastroprotective, antimicrobial, and anti-inflammatory effects

Furthermore, evidence from *in vitro* studies, animal models and clinical trials, suggests that both compound classes may improve cardiovascular health scores, enhance endothelial function, exert gastroprotective effects, reduce blood pressure, and promote antimicrobial activity (Li et al., 2012; McCarty et al., 2015; Irandoost et al., 2021; Romero-Luna et al., 2023; Maharjan et al., 2024). Their anti-inflammatory activity may be useful in counteracting the chronic inflammation associated with obesity and metabolic syndrome (Jolayemi and Ojewole, 2013). However, many of these effects have been demonstrated primarily in preclinical studies or under specific experimental conditions, and their clinical relevance requires further confirmation in well-controlled human trials.

### 3.3 Anticancer properties

Capsaicin demonstrates significant anticancer activity in *in vitro* and animal models by modulating gene expression to induce apoptosis and promote cell cycle arrest in various cancer cell lines. These effects have been documented in multiple cancer types, including colorectal, pancreatic, hepatocellular, prostate, tongue, gastric, and breast cancers (Kim et al., 1997; Jung et al., 2001; Mori et al., 2006; Zhang et al., 2008; Lu et al., 2010; Thoennissen et al., 2010; Pramanik et al., 2011; Lin et al., 2013; Luján-Méndez et al., 2023). Capsaicin’s antiproliferative and pro-apoptotic actions involve disruption of mitochondrial membrane potential and activation of caspases, highlighting its potential as an anticancer agent under experimental conditions. However, these findings should not be interpreted as direct evidence of anticancer efficacy in humans. Large, well-designed clinical studies are still required to validate these effects, establish safe and effective intake levels, and clarify possible differences in individual response (Yang et al., 2009).

### 3.4 Potential adverse effects

Despite their potential benefits, capsaicinoids may cause adverse effects, particularly at high intake levels or in sensitive individuals, including gastrointestinal discomfort, abdominal burning, abdominal pain, heartburn, and reduced tolerability due to pungency (Patcharatrakul et al., 2020). Capsinoids, being non-pungent or only mildly pungent, may offer better sensory tolerability (Silvester et al., 2019), and available human data indicate that single oral doses of capsinoids can be well tolerated, although their effects may depend on dose, formulation, metabolism, and bioavailability (Bernard et al., 2008). Therefore, breeding strategies aimed at modifying capsaicinoid and capsinoid levels should consider not only nutraceutical potential, but also consumer acceptance and safe use.

## 4 Environmental effect on capsaicinoid and capsinoid production

The pungency of peppers is determined by several factors, including genotype, environment, and the interaction between these factors. Breeding programs have exploited the genetic diversity present within *Capsicum* species to develop cultivars exhibiting a broad spectrum of capsaicinoid concentrations, ranging from highly pungent varieties to cultivars with no pungent properties whatsoever (Devi et al., 2021; Darko et al., 2022). Nevertheless, the impact of environmental factors, such as temperature, on the accumulation of capsaicinoids and capsinoids in *Capsicum* species is intricate and exhibits significant variation across different growing conditions and cultivars. Environmental conditions may affect these traits not only by modifying fruit physiology, but also by modulating the expression of key genes involved in capsaicinoid biosynthesis. For example, drought stress in greenhouse-grown *C. annuum* cultivars ‘Shishito’ and ‘Sapporo’ increased capsaicinoid content in the placental septum and altered the expression of several biosynthetic genes, including *ACL*, *pAMT*, *Pun1*, *CaKR1*, *CaMYB31*, *FAT*, and *KAS I* (Rathnayaka et al., 2021). Similarly, promoter analyses showed that the capsaicin synthase/*AT3* promoter is responsive to light, heat shock, wounding, and capsaicin treatment, while *CaMYB31* has been described as a transcriptional regulator of capsaicinoid biosynthetic genes and is responsive to hormonal and stress-related signals (Kim et al., 2009; Arce-Rodríguez and Ochoa-Alejo, 2017**).**

Generally, moderate increases in temperature, particularly during the night, tend to promote capsaicinoid production, but excessive heat stress can be detrimental (Gurung et al., 2011). This response may be associated with changes in fruit development and in the transcriptional regulation of capsaicinoid biosynthetic genes, although the magnitude and direction of the effect depend on genotype and growth conditions. Light intensity correlates with capsaicinoids levels, with an optimum light intensity for plant growth reported at approximately 1,400 µmol·m-2·s-1 under the experimental conditions described by Gurung et al. (2011). Reducing light intensity can have either a positive or negative impact on capsaicinoid accumulation, depending on the specific *Capsicum* species; on the other hand, high light intensities generally lead to a reduction in capsaicinoid production (Gurung et al., 2011; Jeeatid et al., 2017). These effects may reflect changes in carbon assimilation, fruit metabolism, and the expression of pathway-related genes.

The effect of mineral supplementation (N-P-K) has been shown to promote capsaicinoid production. Various studies have investigated the influence of mineral fertilization on capsaicinoid accumulation. In greenhouse-grown habanero pepper (*C. chinense* Jacq. ‘Habanero Naranja’), weekly applications of nutrient solutions containing 0, 1, 7.5, 15, 22, or 30 mM urea showed that 15 mM nitrogen supported flowering and fruit formation while maintaining high capsaicin levels (Medina-Lara et al., 2008). In contrast, potassium appears to have little to no significant impact on capsaicinoid production (Johnson and Decoteau, 1996; Medina-Lara et al., 2008; Monforte-González et al., 2010). Water stress modulates capsaicinoid production. Moderate water deficit often increases capsaicinoid content, while severe drought or excessive irrigation can reduce it; controlled deficit irrigation has been proposed as a strategy to enhance capsaicinoid levels while conserving water resources (Gurung et al., 2011; Ruiz-Lau et al., 2011). The fruit maturity stage at harvest significantly affects capsaicinoid and capsinoid content. Generally, there is no fixed pattern of production and accumulation for these specialised metabolites within the placenta, as it varies across species and cultivars. Typically, a phase of production and accumulation occurs during fruit maturation; however, before full ripeness, this trend may shift due to both reduced biosynthetic activity (i.e., lower expression of capsaicinoid biosynthetic genes during later ripening stages) and peroxidase activity, which promotes the degradation of these compounds (Bernal et al., 1993b; Fan et al., 2020). For example, in the ‘Chiltepín’, ‘Tampiqueño 74’, and ‘Bhut Jolokia’ genotypes, capsaicinoids begin to accumulate between 10 to 20 days post-anthesis (DPA), peaking at around 40 DPA and then decreasing until 60 DPA (Bernal et al., 1993a; Fayos et al., 2019).

## 5 Construction of a unified QTL map related to pepper capsaicinoids

The genetic regulation of capsaicinoid production in pepper is highly intricate, encompassing over 60 candidate genes involved in two distinct metabolic pathways (Mazourek et al., 2009), as well as numerous transcription factors that regulate the temporal and spatial patterns of gene expression. These structural and regulatory genes are listed in **Supplementary Table 1**, together with the available evidence supporting their involvement in capsaicinoid biosynthesis, including genetic, transcriptomic, and functional validation data where available.

Research on the evolution of pungency has emphasized the importance of gene duplication and neofunctionalization in driving the diversification of key genes involved in capsaicinoid biosynthesis, such as *Kas*, *COMT*, and *Pun1*. It is evident that these processes have played a crucial role in enhancing capsaicinoid production. Breeding efforts have increasingly focused on manipulating capsaicinoid and capsinoid levels, driven by interest in developing varieties with specific pungency profiles and health-promoting properties (Jang et al., 2021; Barik et al., 2022). Quantitative trait locus (QTL) mapping and genome-wide association studies (GWAS) have proven instrumental in elucidating genomic regions associated with capsaicinoid and capsinoid accumulation. To gather information on quantitative trait loci (QTLs) deriving from multiple studies related to capsaicinoids, a detailed review of the literature was conducted (**Table 2**), with the goal of serving as a useful resource for understanding the genetic factors behind this trait.

**Table 2:** Article reporting QTLs for capsaicinoids used in the review.

Reference	Method (population)	Reference genome
Rodríguez-Maza et al., 2012	QTL mapping (F2)	NA
Nimmakayala et al., 2016	GWAS	CM334 v1.55
Haile et al., 2023	GWAS	PI159236 v1.2
Kim et al., 2022	GWAS	Dempsey
Lee et al., 2016	QTL mapping (F2)	NA
Reddy et al., 2014	Gene. Mapping (Collection)	CM334 v1.55
Nimmakayala et al., 2014	QTL mapping (DH)	NA
Ben-Chaim et al., 2006	QTL mapping (F2/F3)	NA
Blum et al., 2003	QTL mapping (F2)	NA
Han et al., 2018	GWAS	CM334 v1.55
Wu et al., 2019	GWAS	Zunla-1
McLeod et al., 2023	GWAS	CM334 v1.6

**Figure 2:**
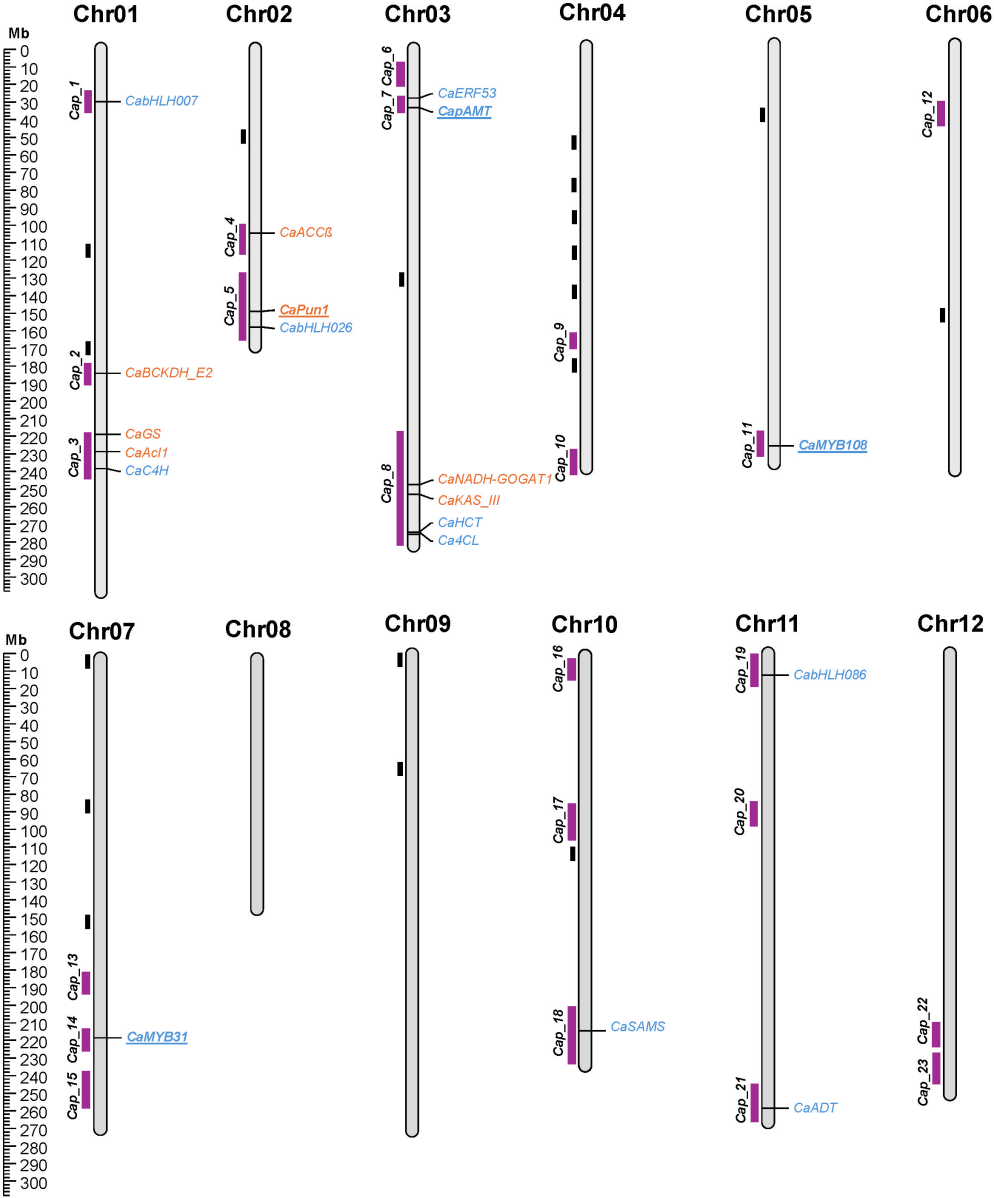
Capsaicinoids QGRs chromosome map (Chr01-Chr12) obtained from the reported literature and its confidence intervals (± 5Mb). Purple bars: QTL Genomic Regions (QGRs). Black bars: QTLs not associated in QGRs. In blue: genes involved in the phenylpropanoid pathway and gene regulations, in orange: genes involved in the fatty acid pathway and Acyl group biosynthesis. Underlined genes: already validates genes involved in capsaicinoids biosynthesis.

In this review, three hierarchical levels of genetic relevance were considered. QTLs correspond to loci originally reported in individual mapping or association studies. By integrating physically mapped QTLs with overlapping or closely adjacent (± 5 Mb) positional intervals on the CM334 v1.6 reference genome, quantitative genomic regions (QGRs) were defined. To ensure the results could be compared across studies and correctly lifted on the reference genome CM334 v1.6 (Kim et al., 2014), only QTLs with known genomic position were included. After data retrieval, QTL information were clustered into Quantitative Genomic Regions (QGRs) in a unified physical map (**Figure 2**). A QGR represents QTLs with overlapping confidence intervals (see below). Candidate genes were then selected among genes located within each QGR, with priority given to genes previously implicated in capsaicinoid or capsinoid biosynthesis, genes belonging to the phenylpropanoid or branched-chain fatty acid pathways, and genes with available genetic, transcriptomic, or functional evidence supporting their involvement in pungency-related traits.

The dataset provides details such as the pathways involved, the genomic regions (QGRs), original QTL names, chromosomal locations, marker types, their positions in centimorgans (cM) and base pairs (bp). It also includes, where possible, statistical results like LOD scores and p-values, the percentage variation explained by each QTL (PVE), the effect of each QTL, the populations or panels used for mapping, and references to the original studies (**Supplementary Table 2**). To address the absence of reported, experimental specific, genetic confidence intervals or the average decay of linkage disequilibrium (LD) for the collected QTLs, positional uncertainties were standardized using an empirically defined window of ±5 Mb around each QTL. This approach doubles the confidence interval suggested by Martina et al. for *Solanum lycopersicum* (Martina et al., 2021). The simple extension of the previously used interval, not based on the LD in the species, appears to be reasonable by the high abundance of transposable elements in the pepper genome, which has resulted in a fourfold increase of the genome size relative to that of tomato (Park et al., 2011).

The different studies were chosen according to the availability and accessibility of the markers; four out of the twelve studies originated from the pre-genomics era when the technological advancements available today were not yet in use. Consequently, only the linkage map positions (cM) were available, and the physical positions of the markers associated with their respective QTLs were not directly accessible. To overcome this limitation, a BLASTn (Altschul et al., 1990) alignment was performed on the CM334 v 1.6 reference assembly (Kim et al., 2014).

### 5.1 Phenylpropanoid pathway genes and regulatory elements

Many of the genes found in QGRs are major pathway genes belonging to the phenylpropanoid pathway. They also provide the necessary precursors to biosynthesize capsaicinoids and capsinoids (**Figure 2**; in blue).

As shown in **Table 3**, in the QGR CAP3, the *C4H* gene (*cinnamate-4-hydroxylase*), *a cytochrome P450-dependent monooxygenase*, catalyzes the reaction that converts cinnamate into p-coumarate. This is a distinguishing intermediate in the biosynthesis of capsaicinoids, flavonoids, capsinoids and lignin (Schilmiller et al., 2009). CAP7 contains the *pAMT* (*putative aminotransferase*) gene, one of the most critical genes in the capsaicinoid biosynthetic pathway, which facilitates the conversion of vanillin into vanillylamine (Kusaka et al., 2024). CAP8 contains two additional genes involved in this pathway: *4CL* (*4-coumarate-CoA ligase*), which activates cinnamic acid derivatives such as caffeic acid by forming a CoA thioester and *HCT* (*hydroxycinnamoyl transferase*), which catalyze the conversion of 4-Coumaroyl-CoA to 4-Coumaroyl-shikimate and the conversion of caffeoyl shikimate to caffeoyl CoA (Hoffmann et al., 2003; Lavhale et al., 2018). *SAMS* in CAP18 encodes *S-adenosyl-L-methionine synthase*, that catalyzes the synthesis of S-adenosyl-L-methionine (SAM) from methionine, a crucial methyl donor in various metabolic processes (Goto et al., 2002; Roje, 2006). Finally, the QGR CAP21 contains the enzyme *ADT (arogenate dehydratase*), which is part of the phenylalanine biosynthesis pathway and provides additional substrate input (Castro-Concha et al., 2016).

**Table 3:** Summary of transcription-factor and phenylpropanoid-biosynthesis genes found in QGRs, with their positions on the reference genome (CM334 v1.6).

Gene name	Gene ID	Chr	Start (Mbp)	End (Mbp)	QGR	Reference
*CaBHLH007*	scaffold1731.6	1	30,84	30,84	Cap_1	(Nimmakayala et al., 2014)
*C4H*	scaffold1731.6	1	239,45	239,47	Cap_3	(Kim et al., 2022; Haile et al., 2023)
*CabHLH026*	scaffold1061.22	2	158,79	158,80	Cap_5	(Rodríguez-Maza et al., 2012; Lee et al., 2016; Haile et al., 2023; McLeod et al., 2023)
*pAMT*	scaffold339.12	3	34,10	34,09	Cap_7	(Kim et al., 2022)
*CaERF53*	scaffold862.63	3	28,66	28,66	Cap_7	(Kim et al., 2022)
*4CL*	scaffold1008.26	3	275,60	275,60	Cap_8	(Ben-Chaim et al., 2006; Lee et al., 2016; Kim et al., 2022; Haile et al., 2023; McLeod et al., 2023)
*HCT*	scaffold1008.1	3	275,26	275,27	Cap_8	(Ben-Chaim et al., 2006; Lee et al., 2016; Kim et al., 2022; Haile et al., 2023; McLeod et al., 2023)
*CaMYB108*	scaffold305.53	5	229,00	229,00	Cap_11	(Kim et al., 2014)
*CaMYB31*	scaffold898.7	7	217,68	217,69	Cap_14	(Blum et al., 2003)
*SAMS*	scaffold900.24	10	215,79	215,80	Cap_18	(Nimmakayala et al., 2016; Han et al., 2018; Wu et al., 2019; Kim et al., 2022; McLeod et al., 2023)
*CabHLH086*	scaffold308.41	11	66,08	66,08	Cap_19	(Nimmakayala et al., 2016)
*ADT*	scaffold919.8	13	259,24	259,24	Cap_21	(Nimmakayala et al., 2016; Han et al., 2018)

The capsaicinoid and capsinoid biosynthetic pathways are subject to a complex regulatory system that involves numerous transcription factors (TFs). A significant proportion of these TFs are present in different QGRs that have been identified in this work. For example, in CAP1, the transcription factor *CaBHLH007* works with other transcription factors from the same family to activate *CaMYB31* (Liu et al., 2021). Similarly, *CabHLH026* regulates pungency and activates *CaMYB31* expression in CAP5 (Liu et al., 2021). In CAP7, *CaERF53*, an ethylene-responsive factor, has been positively associated with capsaicinoid biosynthesis (Song et al., 2020). Two additional MYB transcription factors found across different regions are: *CaMYB108* in CAP11, whose expression positively correlates with capsaicinoid accumulation (Sun et al., 2020), and *CaMYB31* in CAP14, which is considered the key MYB regulator in the capsaicinoid biosynthetic pathway. *CaMYB31* acts as a master regulator by binding to the promoters of multiple genes encoding pathway enzymes, thereby upregulating their transcription (Arce-Rodríguez and Ochoa-Alejo, 2017; Zhu et al., 2019). *CabHLH086* in CAP19, like the other *bHLH* transcription factor, is thought to enhance the activity of *CaMYB31* within the phenylpropanoid regulatory network (Liu et al., 2021).

Genes acting at branch-point steps of the pathway may strongly affect capsaicinoid and capsinoid accumulation because they influence precursor allocation between alternative products. In this pathway, *pAMT* represents a key branch-point gene: when its function is reduced or lost, vanillylamine formation from vanillin is reduced or abolished, while vanillin can be redirected toward vanillyl alcohol, the direct precursor of capsiate and other capsinoids (Lang et al., 2009; Sano et al., 2022). In parallel, QGRs containing transcription factors may explain quantitative phenotypic variation in capsaicinoid accumulation by affecting the coordinated expression of multiple pathway genes. MYB, bHLH, WRKY, and ERF transcription factors have been associated with the regulation of capsaicinoid biosynthetic genes and may contribute to differences in metabolite accumulation among genotypes (Arce-Rodríguez and Ochoa-Alejo, 2017; Zhu et al., 2019; Sun et al., 2020; Liu et al., 2021; Wen et al., 2022). These distinctions are important for breeding, because QGRs containing structural genes may be useful for selecting major capsinoid or pungency-related phenotypes, whereas QGRs containing regulatory genes may provide targets for fine-tuning capsaicinoid levels.

### 5.2 Fatty acid pathway and acyl group biosynthesis genes

Many genes are involved in fatty acid biosynthesis and the acylation steps that are essential for the formation of capsaicinoids and capsinoids (**Figure 2**; in orange). This branch provides the acyl moiety that converges with the phenylpropanoid derived aromatic moiety to complete capsaicinoid and capsinoid biosynthesis. In capsaicinoid formation, the fatty acid-derived acyl donor is condensed with vanillylamine, whereas in capsinoid formation it is esterified with vanillyl alcohol (Lang et al., 2009; Kobata et al., 2013; Sano et al., 2022). In the QGR CAP2, the *BCKDH_E2* gene encodes the E2 subunit of the branched-chain alpha-keto acid dehydrogenase complex, which converts α-ketoisovalerate into isobutyryl-CoA, an important precursor in acylation reactions (Mazourek et al., 2009). In CAP3, *GS* (*glutamine synthetase*) catalyzes the conversion of glutamine to glutamate, providing nitrogen for biosynthetic reactions (Tecson et al., 2025). The same QGR contains the *Acl1* gene (*ATP-citrate lyase*), which converts citrate into cytoplasmic acetyl-CoA, an important substrate for fatty acid biosynthesis (Chypre et al., 2012). In CAP4, *ACCβ* encodes the β subunit of *acetyl-CoA carboxylase*. Together with other enzymes, it catalyzes the transformation of acetyl-CoA to malonyl-CoA, which is the first committed step in fatty acid biosynthesis (Sasaki and Nagano, 2004). CAP8 contains the *KASIII* gene, which is involved in fatty acid chain elongation (Nofiani et al., 2019). The *NADH-GOGAT* enzyme in CAP8 catalyzes the formation of glutamate from α-ketoglutarate, returning additional nitrogen to the fatty acid metabolic system (Suzuki and Knaff, 2005). Finally, *Pun1*, an *acyltransferase* located in the QGR CAP5, adds an acyl group to vanillylamine in the final step of capsaicinoid formation (Han et al., 2013) (**Table 4**). This terminal acyltransferase reaction represents a key biochemical step in capsaicinoid production, mediating the convergence of the fatty acid-derived acyl donor and the phenylpropanoid-derived vanillylamine acceptor (Ogawa et al., 2015). Thus, the QGR CAP5, harboring *Pun1*, may be particularly relevant for interpreting pungency-related phenotypic variation.

**Table 4:** Summary of fatty acid pathway and acyl group biosynthesis genes found in QGRs, with their positions on the reference genome (CM334 v1.6).

Gene name	Gene ID	Chr	Start (Mbp)	End (Mbp)	QGR	Reference
*BCKDH_E2*	scaffold1137.1	1	185,22	185,21	Cap_2	(Haile et al., 2023)
*GS*	scaffold522.8	1	219,87	219,87	Cap_3	(Kim et al., 2022; Haile et al., 2023)
*Acl1*	scaffold223.5	1	232,13	232,14	Cap_3	(Kim et al., 2022; Haile et al., 2023)
*ACCβ*	scaffold1478.9	2	105,48	105,49	Cap_4	(Reddy et al., 2014)
*PUN1*	scaffold809.56	2	150,11	150,12	Cap_5	(Rodríguez-Maza et al., 2012; Kim et al., 2022; Haile et al., 2023; McLeod et al., 2023)
*KASIII*	scaffold732.11	3	253,80	253,82	Cap_8	(Ben-Chaim et al., 2006; Lee et al., 2016; Kim et al., 2022; Haile et al., 2023; McLeod et al., 2023)
*NADH-GOGAT*	scaffold108.62	3	246,38	246,40	Cap_8	(Ben-Chaim et al., 2006; Lee et al., 2016; Kim et al., 2022; Haile et al., 2023; McLeod et al., 2023)

## 6 Future perspectives

The genetic basis governing the synthesis of both capsaicinoids and capsinoids has been extensively studied and well-documented over the past few decades, owing to their unique occurrence in *Capsicum* spp. and their significance as both food components and health-related bioactive molecules. However, the information generated from these studies is often difficult to compare. This is due to several factors, such as the different types of mapping populations used, the number of markers, the evolution of sequencing techniques, and the availability of multiplegenome assemblies in pepper, which has resulted in the utilization of different genome references in gene mapping studies.

The primary aim of this review was to consolidate and compare findings from diverse studies, each employing different methodologies, and unify them into a comprehensive analysis. Overall, 23 distinct QGRs from twelve different studies, defined as genomic regions most likely to contain genetic elements regulating capsinoids and capsaicinoids presence in pepper, were identified. These QGRs have been found to harbor nineteen genes representing two pathways involved in capsaicinoid and capsinoid production. Some of these genes have already been validated, including *Pun1* in Cap_5*, pAMT* in Cap_7, *CaMYB108* in Cap_11 and the master regulator *MYB31* in Cap_14 (del Rosario Abraham-Juárez et al., 2008; Arce-Rodríguez and Ochoa-Alejo, 2015, 2017; Sun et al., 2019). The information on these QGRs and the markers used to build them provides a unified resource for breeders aiming to develop superior germplasm with a high organoleptic and nutraceutical value related to controllable and stable levels of capsaicinoids and capsinoids through marker-assisted selection (MAS). In practical terms, these regions may support the selection of parental lines, the introgression of favorable alleles, and the development of cultivars with more controllable and stable levels of capsaicinoids and capsinoids. Additionally, the identified potential candidate genes can be further investigated by researchers through transient manipulation of gene expressions to evaluate their specific effects; in parallel, these candidate genes represent valuable targets for functional validation and for evaluating their specific contribution to capsaicinoid and capsinoid biosynthesis.

Nevertheless, the practical application of QGRs should take into account the influence of environmental and developmental factors on capsaicinoid and capsinoid accumulation. The expression of pungency-related traits may vary depending on growing conditions, fruit developmental stage, and genetic background. Therefore, QGRs and associated markers should be validated across additional populations, environments, and developmental stages before being routinely applied in breeding pipelines. Overall, this review provides an accessible and comparative framework that can improve the reliability of QTL interpretation, guide candidate gene prioritization, and facilitate the translation of genomic information into MAS strategies for the improvement of capsaicinoid and capsinoid profiles in pepper.
